# Supplementary material for: Pain assessment in horses using automatic facial expression recognition through deep learning-based modeling
Source: PLoS One. 2021 Oct 19;16(10):e0258672. doi: 10.1371/journal.pone.0258672 (PMC8525760; doi:10.1371/journal.pone.0258672)
Supplement: Attachment — Submitted filename: Response to Reviewers.docx [file pone.0258672.s001.docx]

Response to Reviewers

Dear Dr. Humaira Nisar and reviewers,

Thank you very much for your comments. They were extremely helpful in order to improve the quality of our manuscript.

In the document below we are describing how we have answered the questions and how we did carry out the necessary corrections to the manuscript.

1. Please ensure that your manuscript meets PLOS ONE's style requirements, including those for file naming. The PLOS ONE style templates can be found at

<https://journals.plos.org/plosone/s/file?id=wjVg/PLOSOne_formatting_sample_main_body.pdf> and

<https://journals.plos.org/plosone/s/file?id=ba62/PLOSOne_formatting_sample_title_authors_affiliations.pdf>

We have reviewed the manuscript based on the PLOS ONE´s style requirements.

2. In your Methods section, please provide additional details regarding participant consent from the owners of the animals. In the ethics statement in the Methods and online submission information, please ensure that you have specified (1) whether consent was informed and (2) what type you obtained (for instance, written or verbal). If the need for consent was waived by the ethics committee, please include this information.

We have added this information to our Methods section on the manuscript. (Lines 136 – 139).

3.In your Data Availability statement, you have not specified where the minimal data set underlying the results described in your manuscript can be found. PLOS defines a study's minimal data set as the underlying data used to reach the conclusions drawn in the manuscript and any additional data required to replicate the reported study findings in their entirety. All PLOS journals require that the minimal data set be made fully available. For more information about our data policy, please see <http://journals.plos.org/plosone/s/data-availability>.

Our data are available in the Mendeley Repository: (Lencioni, Gabriel; Sousa, Rafael; Sardinha, Edson; Romero, Rodrigo; Zanella, Adroaldo (2021), “Automatic Pain Assessment in Horses”, Mendeley Data, V1, DOI: 10.17632/t8rtzcgwxm.1).

Upon re-submitting your revised manuscript, please upload your study’s minimal underlying data set as either Supporting Information files or to a stable, public repository and include the relevant URLs, DOIs, or accession numbers within your revised cover letter. For a list of acceptable repositories, please see <http://journals.plos.org/plosone/s/data-availability#loc-recommended-repositories>. Any potentially identifying patient information must be fully anonymized.

Our data are available in the Mendeley Repository: (Lencioni, Gabriel; Sousa, Rafael; Sardinha, Edson; Romero, Rodrigo; Zanella, Adroaldo (2021), “Automatic Pain Assessment in Horses”, Mendeley Data, V1, DOI: 10.17632/t8rtzcgwxm.1).

Important: If there are ethical or legal restrictions to sharing your data publicly, please explain these restrictions in detail. Please see our guidelines for more information on what we consider unacceptable restrictions to publicly sharing data: <http://journals.plos.org/plosone/s/data-availability#loc-unacceptable-data-access-restrictions>. Note that it is not acceptable for the authors to be the sole named individuals responsible for ensuring data access.

We will update your Data Availability statement to reflect the information you provide in your cover letter.

4.Thank you for stating the following financial disclosure:

"The funders had no role in study design, data collection and analysis, decision to publish, or preparation of the manuscript."

At this time, please address the following queries:

a. Please clarify the sources of funding (financial or material support) for your study. List the grants or organizations that supported your study, including funding received from your institution.
b. State what role the funders took in the study. If the funders had no role in your study, please state: “The funders had no role in study design, data collection and analysis, decision to publish, or preparation of the manuscript.”
c. If any authors received a salary from any of your funders, please state which authors and which funders.
d. If you did not receive any funding for this study, please state: “The authors received no specific funding for this work.”

Please include your amended statements within your cover letter; we will change the online submission form on your behalf.

5. Please upload a copy of Figure 5, to which you refer in your text on page 18. If the figure is no longer to be included as part of the submission please remove all reference to it within the text.

In the manuscript there is no Figure 5 included. All the references pointing to it on the text were removed.

**Additional Editor Comments:**

The manuscript aims to develop an automated method for the detection of post-operation pain of horses using convolutional neural networks.

The topic and the work is very interesting.

However reviewers have major concerns about the technical aspects of the paper, as many important parameters have not been explained appropriately. Which is probably because of the reason that the paper writing also needs a major improvement.

The reviewers have given detailed comments which will be very useful in improving the quality of this manuscript. Hence based on the reviewers recommendation i will recommend a major revision.

**Comments to the Author**

1. Is the manuscript technically sound, and do the data support the conclusions?

The manuscript must describe a technically sound piece of scientific research with data that supports the conclusions. Experiments must have been conducted rigorously, with appropriate controls, replication, and sample sizes. The conclusions must be drawn appropriately based on the data presented.

Reviewer #1: Partly

Reviewer #2: Partly

Reviewer #3: Partly

2. Has the statistical analysis been performed appropriately and rigorously?

Reviewer #1: Yes

Reviewer #2: No

Reviewer #3: N/A

3. Have the authors made all data underlying the findings in their manuscript fully available?

The [PLOS Data policy](http://www.plosone.org/static/policies.action#sharing) requires authors to make all data underlying the findings described in their manuscript fully available without restriction, with rare exception (please refer to the Data Availability Statement in the manuscript PDF file). The data should be provided as part of the manuscript or its supporting information, or deposited to a public repository. For example, in addition to summary statistics, the data points behind means, medians and variance measures should be available. If there are restrictions on publicly sharing data—e.g. participant privacy or use of data from a third party—those must be specified.

Reviewer #1: Yes

Reviewer #2: No

Reviewer #3: Yes

4. Is the manuscript presented in an intelligible fashion and written in standard English?

PLOS ONE does not copyedit accepted manuscripts, so the language in submitted articles must be clear, correct, and unambiguous. Any typographical or grammatical errors should be corrected at revision, so please note any specific errors here.

Reviewer #1: Yes

Reviewer #2: No

Reviewer #3: No

**Reviewer #1: 0.**

The manuscript presents and discusses the practical applicability of CNN for the automated detection of post-operation(castration) pain of horses. The work presented involved the design of a shallow 2-conv-layer CNN for the classification of manually cropped images of horse faces, extracted from videos recorded before and after the castration surgery. The importance and relevance of the work are properly outlined in the introduction. The work reported a significant performance enhancement with the ensemble prediction using voting system, compared to individual CNN trained on a single face region of interest (ROI).

Nevertheless, the manuscript seems written in a hurry. Expression of sentences were not properly proof-read by the authors prior to submission.

Thank you for all the observations, they were very helpful during our editing process.

The manuscript has now been reviewed by a native English speaker which we believe has helped improve the readability of the text.

For example, in Lines 101-108 there are duplication and badly organized phrases. Lines 133-137 are of different font than the other paragraphs.

Apologies, the text has been corrected.

However, there could still be valuable research contributions with following questions addressed, which have arised in the review process. In addition, the HGS-labelled image dataset generated in this study could potentially chip in to enhance the variation of publicly available HGS dataset for research.

The dataset generated in this study is available in the Mendeley data repository as we agree that the lack of datasets, that could be used for studying pain in animals is very limiting for robust studies. We do hope that these data will facilitate data sharing.

Question 1:

Lines 126-130:

How do 7 horses, for (2+4) days, for 4 times a day, result in 1252 videos?

(7 horses x 6 days x 4 times/day = 168 videos) Were multiple recording cameras used for every horse?

This information has been corrected in the manuscript (lines 128-130)

The actual number is 320 videos of 30 minutes duration each. (7 horses x 6 days x 4 times/day (more than a video per time point since the videos had a maximum length of 30 minutes). Thank you for pointing out our mistake.

Question 2:

It is commendable and helpful for reviewing that the authors have clearly provided the details like the CNN architecture, CNN kernel size, and the width and height of input images in Table 2 and Table 3.

Could the author explain why shallow CNN of only 2 convolutional layers were used? Couldn't a few additional convolutional layers benefit the feature extraction and abstraction capability of the model as the input are raw images and the output demanded is a highly abstract emotional expression of animal?

Indeed, we consider it important to show the configurations and final architecture so that the method can be replicated.

We agree that the more convolutional layers the better, but as convolutional layers are added, the number of input resources for subsequent layers is reduced and the processing demand increases significantly. Our goal is to show the method's viability, that's why we haven't advanced in testing other architectures. We hope that our methods can inspire future work that explores other architectures.

Question 3:

It is again commendable that authors honestly and clearly reported the low Recall performance for the presence of pain (esp. for the obvious pain) in each facial subcategory. This is important as the low recall indicates the failed detection of obvious pain which is of greater concern in pain management. This may reflect the insufficience of each individual facial category to serve in pain detection by the presented CNN model.

Has the author not considered increasing the reliability of HGS with independent labelling performed by more than only one trained-observer (as reported in line 159)?

Indeed, the analysis of the performance in each facial subcategory is relevant.

We have considered having more trained observers performing the labeling and we understand that this can improve the overall accuracy of the method. We have discussed this information in the text (Lines 304 – 307). However, we plan to carry out work, on this area, on for future research, since in this study our aim was to evaluate if this method would be useful. Now that we have this initial answer it is possible for us and other scientists to improve the details of the system, and perhaps exchange information and create solid partnerships.

Question 4:

For contents in Lines 202-207 & Lines 252-259:

Based on which image pool was the voting/ensemble model weight-tuned? Was it using the image pool that each member CNN of the ensemble model was trained on (as that presented in Table 1 & 3) or another separate image pool? Or was the weight-tuning also based on the pool of 30 images reported in Table 7 and Table 8 (which shouldn't be the case as the tuning process should not have access to the final test set)? The authors has not explicitly reported the image dataset used for the ensemble/voting weight-tuning process.

If this ensemble voting model with weight ratio of 3:1:1 for eye:ears:mouth&nostril is applied back onto the very original image dataset presented in Table 1 before these images are cropped into ROI, what is the performance of the model is terms of accuracy, precision and recall?

The weight tuning process (which is a key step resulting in the pain classification performance surge) should be analyzed and discussed in greater details.

What is shown in Table 1 is the number of cutouts made in original complete images. Automatic pain classification on the original image has not been implemented. This requires the development of another method (additional algorithm), in which the automatic search for the clippings of interest in the original image or video would be implemented, and then the proposed method would be applied. In our case, this process was performed manually, but for a final automatic system this development would be necessary.

Thank you for your observation, we agree that the voting model needed to be explained in more details. We have added explanation of the voting process, weight adjustments and image selection in the manuscript, (lines 207 – 216).

**Reviewer #2:** The paper aims at employing artificial intelligence in identifying the existence of pain and its level in horses. Although the problem addressed by the paper is exciting and carries great future potential in opening research avenues, the paper still needs much work to be publication worthy. I have the following concerns:

1. The writing of the paper needs revision. There are many repetitions that should have been omitted after the authors decided on the final shape of the paragraph. The first paragraph of the data collection section (starting line 101) is a prime example. These are not style mistakes but rather genuine writing/editing flaws. See line 58.

Thank you for your observations. These corrections were important and have been done. Also, the manuscript has now been reviewed by a native English speaker.

2. The authors need to enroll a computer science specialist with knowledge in AI. She/he will contribute to better experiments and to writing in the proper computer science jargon. This is a problem with the current manuscript as it is written by non-specialists.

The presented work is strongly multidisciplinary and our challenge was to create a report that could communicate the proposal and the results to scientists from different areas, such as computer science, veterinary and animal science. I would like to count on your understanding for this challenge since one of our aims is to show for other animal scientists and veterinarians how it is possible to have amazing outcomes when associating knowledge from the animal health area with computer science technology.

3. The data collection section, details of the drugs given are not necessary for the contribution of the paper (unless they are required for ethical approval).

Although the details of the drugs given may not be necessary from the perspective of the system development, we believe that this information could be relevant for other scientists working in the veterinary and animal science areas as it could interfere in the expression of pain.

4. Section III.II, the number of images 185.000 should be written as 185000, and there is no need for approximately as this is a simple count. The exact number can be easily included.

The information has been corrected in the manuscript (lines 141 – 143).

5. The authors need to elaborate more on the time and effort to inspect 185000 images to select 3000 of them (if not randomly). This does not seem right.

A computer program was used that, based on motion detection, selected frames in the videos. After this process, images with quality and representativeness of each pain level were manually selected, in order to select only the ones that made the pain evaluation possible. It was a very demanding task that took months of work, but we have managed to do it. We attempted in the text to clarify the information.

6. The approach to select the proper images could be better than just inspecting the individual frames, which carry a lot of repetitions especially at 30 frames per second video rate.

As answered in the previous question, in order not to have to inspect all the frames captured by the camera, we have used an application software that extracted frames only when motion was detected (We have added this information to the manuscript, line 141 -145). Then, the resulting frames were selected in order to get only the ones that were possible to evaluate pain through facial expressions. Of course, this is still a very demanding process, however we wanted to make sure that we got all the images possible in order to result in a good image database.

7. In general, the AI approach need to be driven by a computer scientist so that many of the issues in this paper could be ironed out. For example, the number of subjects (i.e., 7) is too small and may lead to overfitting of the model.

We agree that having more subjects would be ideal. Between these 7 seven subjects that we managed to include in this study, we had animals with different coat colors and different breeds, in order to improve the variability in the facial characteristics that the system would interpret. However, our proposal is novel and our goal with dissemination is to show its potential, and thus inspire other groups of scientists who can develop the work further. Right now, we have already been working on future developments for models with more subjects and a larger database. However, these ongoing improvements would benefit from the successful results that we would like to have feedback, from the current study.

8. The font size in paragraph on line 133 need to be consistent with the paper (such mistakes reflect badly on the quality of the paper).

It was corrected, thank you.

9. It is not clear how the system combines the result from the three body parts to form a judgement on pain. The current approach seems overly simple.

Thank you for your observation, we agree that the voting model needed to be explained in more details. We have added explanation of the voting process, weight adjustments and image selection in the manuscript, (lines 207 – 216).

10. Numbering of sections need to better.

We have reviewed the manuscript based on the PLOS ONE´s style requirements and corrected it.

**Reviewer #3**: The manuscript ‘Pain assessment in horses using automatic facial recognition through deep learning-based modeling’ is an interesting and timely manuscript. The results seem indeed promising to be able to record facial expressions of pain automatically and this is clearly worthwhile for practice.

However, I believe some aspects need to be addressed with more detail and there are a couple of things I do not understand which may need clarification first before the manuscript may be accepted for publication in PLOS ONE.

Below, you will find my arguments for the answers I have given to the questions that are part of the PLOS ONE review process and additional detailed feedback.

Title: I am not sure recognition is the right word to use. To me facial recognition would be that a computer can identify animal 1 from 2 or can tell this is an ear or a mouth. But in this study the assessment was aimed to say that if the ear was positioned like this, there is no pain present. That seems to be something different?

Thank you for these observations, they were very helpful to improve the manuscript.

We totally agree with this aspect related to the title. We have corrected the title for: Pain assessment in horses using automatic facial expression recognition through deep learning-based modeling.

Abstract

Line 2: ‘The aim of the study was ....’ instead of ‘The aim of the study is ....’?

The text was corrected.

Lines 6 (and 60): Yes, clear that training is important for using the HGS. However, a computer model needs to be trained too and if this is done not properly, it does not really help us forward. So not sure to what extend a computer model is an advantage then?

To create models based on supervised machine learning, training is required. However, after the construction of the final model (final algorithm), it can be used in any place indefinitely, being part of a computer vision system. On the other hand, when we are training people to apply the pain scale, we end up depending on their availability to evaluate the animal, which will not be the same as the system that could work full time, without supervision.

Lines 8 (and 64-66): True, but a person may not always be needed to be present? The animal can also be monitored from cameras for instance. So this is not always a problem for evaluation, I think.

We agree that a person's evaluation through a camera won't lead to a possible bias to the animal altering its pain expression due to a threatening stimulus. However, the idea is to create an automatic system that doesn't depend on human availability, since it would require large amounts of time to constantly evaluate each animal from time to time. The system would be able to do this task uninterruptedly.

Line 15: ‘process was applied’ and ‘methods were used’ instead of ‘.... is’ and ‘.... are’? May apply at other sentences too.

It was corrected.

Introduction

Line 28: [1,2] instead of [1][2]

It was corrected.

Lines 33 and 56: A space is missing before the reference. Please check whether a space is missing at other places perhaps too.

It was corrected.

Line 55: The word ‘if’’ is not needed, I think.

It was corrected.

Lines 59, 105 and 174: I think here is one space too many. Please check whether an extra space is present at other places perhaps too.

It was corrected.

Line 77: Full stop is missing after the references.

It was corrected.

Materials and Methods

Lines 88 and 92: I am not sure I understand what the authors mean with application exactly. To be able to reproduce the study, more details on this method may be needed to give.

It was complemented in the manuscript that “application” is referring to a “application software”. Line 86.

Lines 101-105: Please check these lines as things are said twice here.

It was corrected.

Line 125: What was the total length of the castration procedure? May be good to add to show the reader it was done as quickly as possible.

Thank you for your observation, we agree that this information is an important concern. The total length of the procedure was approximately 40 minutes (time considering anesthesia and recovery) including 20 minutes of the surgery procedure itself.

This information was added to the manuscript (lines 123 – 124).

Line 130: ‘videos of 30 minutes’ instead of ‘videos with 30 minutes?

It was corrected. (Line 129).

Line 130: With 1252 videos, this means you have about (1252 / 7 horses / 6 days / 4 time points =) 7 videos with a total of 3.5 hours of footage around a time point? That seems sufficient, I am just wondering how to visualize this so to say as there is for example only 2 hours in between time point 10 am and 12 pm. When was a video then considered to belong to a certain time point? Or do I not understand this correctly?

This information has been corrected in the manuscript (lines 128-130)

The actual number is 320 videos of 30 minutes duration each. (7 horses x 6 days x 4 times/day (more than a video per time point since the videos had a maximum duration of 30 minutes).

Lines 133-137: Seems to be in a different letter font?

It was corrected.

Lines 135 and 137: May be good to add when exactly on a day this was done, as I think it should not have interfered with making the videos of Line 130?

Post-castration follow ups were carried out from 07:00 am. Images were never collected when the presence of humans was recorded. It is possible that the images were collected and used for the study after the evaluations were carried out, however this should not have interfered with the measures obtained. Thank you for the useful question.

We have added this information to the manuscript (lines 134 – 136).

Lines 147-148: What is exactly part of the 4th and 5th parameter? You have to guess a bit where the 4th ends and the 5th begins.

We agree that this was not clear in the manuscript. We have corrected it (Lines 146 – 148).

Line 157/Fig. 2: I would like to suggest to take another picture as example for the mouth and nostrils parameter for moderately present. As I do not really understand what I am looking at here, I am afraid. If possible, a more clear one as used for not present and obviously present would definitely help.

In the picture of pain moderately present for the Mouth and nostrils parameter it is possible to notice that, in comparison to the pain not present image for the same parameter, the line between upper and lower lip (mouth column) is shorter, (but not as short as in the pain obviously present example). The mouth is slightly strained and so are the nostrils. However, we agree that this interpretation can be quite difficult, so we also added this information to the figure 2 legend in the manuscript. (Lines 157 – 161).

Lines 159-161: Was the pain assessment solely based on the HGS? Or also based on other behavioral or physiological parameters? In other words, how did the authors know the horse was really in pain? The horses were given post-operative analgesia, so perhaps they did not feel so much pain? For training and testing a computer model this may not be such an issue, but for practice it is. The ears, for instance, could also be turned backwards due to focusing on a sound and not related to pain, and I think that such false warnings to the veterinarian or horse owner is not desired. Or it is the case that only if all three parameters are scored as pain present by the model, a warning is given?

The reviewer's remarks are important. This is an initial work that seeks to show the potential of the proposed method. This method would only notify the presence of pain if there is more than one of the parameters indicating that state, therefore minimizing false warnings due to a change in only one of the parameters, for example the ears reacting to sound. The study was based solely on the published and validated HGS (Dalla Costa, 2014), and the question was related to the ability of a computer system to detect the changes. For a diagnosis to implement a treatment schedule, for example, threshold for responses could be used.

Lines 165-166: I am sorry, but I do not understand how the authors can have a final database of 4847 images if 3000 were selected in the first place? In addition, summing up 2379, 1436 and 1035, I get 4850 images. Somethings seems to be not entirely right here.

Each one of the original 3000 images of the horse´s full head has been used to generate image clippings for each one of the parameters, that is why it is possible to have more than 3000 images when adding up all the classes and parameters. We have corrected the mistake in the summing up and also added this explanation to the manuscript since we agree that this was not clear (Lines 169 – 172).

Line 173: ‘were built’ instead of ‘was built’

It was corrected.

Lines 177-181: I am not an expert on this yet, but what I learned is that others used much more images/frames for training (and testing) the model. Please see for instance: <https://arxiv.org/pdf/1909.12605.pdf>; <https://www.nature.com/articles/s41598-020-70688-6>; <https://www.mdpi.com/1424-8220/19/5/1188/htm> Perhaps it depends on what exactly the model needs to be trained in, but it may be good to add here why the authors know/feel these numbers are sufficient.

The reviewer's remarks are important. This is a preliminary work that seeks to show the potential of the proposed method. We have been working now on expanding the image bank to refine the computational model (algorithm), however, we needed to make sure that the system would be successful and promising in order to be able to gather resources for all the possible improvements. We also aim to spread the word to inspire other groups of scientists who can evolve with the work, and create potential collaborative arrangements.

Line 195/Table 2: Fig. 5 is not included in the manuscript?

Apologies, it was corrected. Where it was saying Fig. 5 is actually Table 3. (Line 200 – 203 – Table 2).

Lines 202-203: More details on the voting process would be interesting and relevant to give with respect to be able to reproduce the study.

Thank you for your observation, we agree that the voting model needed to be explained in more detail. We have added explanation of the voting process, weight adjustments and image selection in the manuscript, (lines 207 – 216).

Lines 205-206: ‘Thus, a variety of ... was tested’ instead of ‘Thus, it was tested a variety of .....’ makes more sense to me.

It was corrected.

Results and Discussion

Line 215: I would like to suggest to add ‘obviously’ to this line after ‘being’.

It was corrected.

Line 219: It took me some time to figure out where the numbers in Table 4 came from, until I realized that this was based on the test frames which was 237 images for the ears (10% of the total images). It would be good to repeat that somewhere here, I think. Please, be precise and clear which numbers are total and which used for training, validation and testing as the numbers given in Lines 214-215 are the overall total and not the ones used to get Table 4 and that may give a biased picture to the reader. May apply to Lines 231-233, 236-237 and 244-245 as well.

Thank you for this observation. This information was indeed not clear as it could be. We have added the details to precise the testing dataset used in the manuscript in lines (225 – 229) (241 – 244) (255 – 258).

In addition, are there not any results to mention for the validation part of the study?

Through the process of building models using artificial intelligence, databases are partitioned, with a fraction of the database being reserved for model building and validation and another part for the final performance evaluation. In our case representing 80%, 10% and 10% respectively.

Line 230: I would like to suggest to rephrase ‘on the eye’ as it feels as strange formulating in this way.

It was corrected. Line 238.

Lines 232 and 245; I would like to suggest to rephrase this as ‘moderately pain present, and xx of obviously pain present’, because that is more clear.

It was corrected.

Line 270: I would add ‘clearly’ or ‘obviously’ before ‘in pain’.

It was corrected.

Line 286: ‘in horses’ instead of ‘on horses’

It was corrected.

Line 289: Indeed a larger databank would be worthwhile. Do the authors have suggestions on the amount of images desired here?

There is no definitive answer, further research is needed. We are working to have a bank with 20000 images.

Line 293: Can the authors elaborate a bit more on what this bias would mean if present?

The aim is to minimize the impact of individual evaluation failures by using a larger number of evaluators. This information was added in the manuscript in order to make it more clear in lines (304 – 306).

Line 294: Nice to know the authors have in mind to include more behaviours. Do the authors have specific suggestions for these behaviours?

Our ongoing research at the veterinary teaching hospital has offered the opportunity to gather a large dataset on images obtained from clinical cases. Our goal is to explore further information on level of activity, eating, drinking, among other behaviors. Some behaviors that could also be important to be taken into account when evaluating pain are sleep, posture, facial expressions related to stress or aggression, posture, tail movement and others.

Conclusion

Line 303: Increasing the quality was not mentioned before, or was it? If not, I believe one cannot raise new aspects in the conclusion, so please include the quality in the discussion first as well.

We have included this aspect in the discussion too in line 301.
