# Supplementary material for: Pain assessment in horses using automatic facial expression recognition through deep learning-based modeling
Source: PLoS One. 2021 Oct 19;16(10):e0258672. doi: 10.1371/journal.pone.0258672 (PMC8525760; doi:10.1371/journal.pone.0258672)
Supplement: Attachment — Submitted filename: Response to reviewers.docx [file pone.0258672.s002.docx]

Dear Dr. Humainara Nisar and reviewers,

Thank you very much for your comments, we have addressed all the points with extreme attention and we believe that now the manuscript has improved even more. We would like to thank you again for such important considerations.

In the document below we are describing how we answered the considerations and changes on the manuscript.

Additional Editor Comments:

Based on the reviewers comments, the manuscript is not ready for publication yet.

[Note: HTML markup is below. Please do not edit.]

Reviewers' comments:

Reviewer's Responses to Questions

**Comments to the Author**

1. If the authors have adequately addressed your comments raised in a previous round of review and you feel that this manuscript is now acceptable for publication, you may indicate that here to bypass the “Comments to the Author” section, enter your conflict of interest statement in the “Confidential to Editor” section, and submit your "Accept" recommendation.

Reviewer #1: (No Response)

Reviewer #3: (No Response)

2. Is the manuscript technically sound, and do the data support the conclusions?

The manuscript must describe a technically sound piece of scientific research with data that supports the conclusions. Experiments must have been conducted rigorously, with appropriate controls, replication, and sample sizes. The conclusions must be drawn appropriately based on the data presented.

Reviewer #1: Partly

Reviewer #3: Yes

3. Has the statistical analysis been performed appropriately and rigorously?

Reviewer #1: N/A

Reviewer #3: N/A

4. Have the authors made all data underlying the findings in their manuscript fully available?

The [PLOS Data policy](http://www.plosone.org/static/policies.action#sharing) requires authors to make all data underlying the findings described in their manuscript fully available without restriction, with rare exception (please refer to the Data Availability Statement in the manuscript PDF file). The data should be provided as part of the manuscript or its supporting information, or deposited to a public repository. For example, in addition to summary statistics, the data points behind means, medians and variance measures should be available. If there are restrictions on publicly sharing data—e.g. participant privacy or use of data from a third party—those must be specified.

Reviewer #1: Yes

Reviewer #3: Yes

5. Is the manuscript presented in an intelligible fashion and written in standard English?

PLOS ONE does not copyedit accepted manuscripts, so the language in submitted articles must be clear, correct, and unambiguous. Any typographical or grammatical errors should be corrected at revision, so please note any specific errors here.

Reviewer #1: Yes

Reviewer #3: Yes

6. Review Comments to the Author

Please use the space provided to explain your answers to the questions above. You may also include additional comments for the author, including concerns about dual publication, research ethics, or publication ethics. (Please upload your review as an attachment if it exceeds 20,000 characters)

Reviewer #1: 【Question 1 follow-up】:

Authors' response is accepted. Corrective action has been made.

【Question 2 & 3 follow-up】:

Authors have responded by saying further future work will be done in these directions.

It is okay for Question 3 not to be addressed with corrective research action as it will require almost another full round of research work apart from the video recording.

Thank you for understanding the limitations to address question 3. We are committed to continue this area or research and your comments are certainly valuable to improve our future work.

【Question 4 follow-up】:

According to Table 1, the datasets are highly imbalanced (within the 3 classes: absence of pain, moderate pain, and obvious pain) with vast majority of the images being under absence of pain. So, the focus of performance analysis would naturally be put on the Recall and Precision of the categories with pain (moderate pain and obvious pain). After all, this model is for the purpose of pain assessment.

Using only one of the three facial regions (the ears, eyes, or the nostrils), the system's pain detection Recall performance was from around 27%-67% as recorded in Tables 4, 5, and 6. This high-accuracy low-recall performance could be due to the vastly imbalanced training image pool, with the CNN parameters being tuned to favor the detection of the absence of pain. Perhaps the authors could consider improving the balance of the dataset, or providing counter-measure for this issue.

The balance of the dataset is important especially for the test dataset. Out of the 237 test images in the ear category, 198 of them are under the absence of pain. The model performance tested using such an imbalanced test set is likely not properly reflecting the true accuracy of the model.

It was reported that only 10 random images (out of the total of 3000 selected images) were used for the performance testing of the final ensemble voting model. Are there any valid reasons for why the authors would not allocate a greater amount of images for the final performance test? Just for instance, at a ratio of 6:1:1:1:1 for the following purposes:

- 60% (for individual CNN training):

- 10% (for individual CNN validation):

- 10% (for individual CNN testing):

- 10% (for ensemble voting weight tuning):

- 10% (for final ensemble test)

, instead of using only the same 10 images out of the 3000 for both the voting-weight tuning and final testing.

Thank you for all your relevant considerations, they were really helpful in order to improve the quality of the manuscript. We agree that ideally the classes should have a more balanced design aiming to an even better and more robust outcome for the reported system. We discussed in the manuscript that working with images of animals, especially in pain models, brings the associated challenges to work with small datasets. That is why we chose to use all the images possible for each model, even though this would result in an unbalanced model the training for each was the best that we were able to do. We are developing now on our current research, ways to improve the dataset and as a result we should be able to improve the system with a more balanced model adding more images.

We have made changes in our final algorithm to address the issues raised regarding the number of images and the performance test, which we will discuss in detail in the next question, since it is also related to the tuning process.

Furthermore,

【Page 12. Lines 223-227】

The same images were used for tuning the "voting-weight" ratio and for the final ensembled performance test. This is not a correct practice. By doing so (using the same images for tuning and testing), the reported weight ratio was not properly validated and may not be well applicable onto wider variations of pain expression.

As the authors have responded to my Question 2 & 3, saying that "in this study our aim was to evaluate if this method would be useful" and "hope that our methods can inspire future work that explores other architectures", the main concern may not be on reporting very high classification accuracy. However, it is important to present an analysis report that is technically and statistically sound.

We would like to thank the reviewer for the important comments. We were able to make improvements to our system and we believe that now our results are more robust and accurate. As a solution for these concerns, we chose to substitute the voting system for a machine learning method, based on Artificial Neural Network (ANN-based classifier) and a Perceptron feedforward and multi-layered architecture, with a sigmoid transfer function in the hidden layer and a linear transfer function in the output layer. The Levenberg-Marquardt backpropagation method and mean squared error were employed to measure the performance using k-fold cross validation of 10. In addition to that, we have improved our database for the process of training and testing of the classifier, that went from a total of 30 images to 120 images on the new model. With these changes, the overall accuracy of the final model was slightly lower than the previous, however we believe that it was worth in order to address all the topics discussed previously. Again, thank you for your comments

【Additionally】,

For facilitating the review process of the contribution of this paper, it would be helpful to include a systematic literature review on others' previously-published relevant research and their relevant research achievement, in order to present a clearer overall picture of current state of research in this subject of interest. For instance, the automated decoding of expression or pain in horses. Or the automated decoding of expression or pain in other animals, if the reported works on horses are currently too limited.

Recently we came across the article (Andersen PH, Broomé S, Rashid M, Lundblad J, Ask K, Li Z, et al. Towards machine recognition of facial expressions of pain in horses. Animals. 2021;11. doi:10.3390/ani11061643) that offers a comprehensive review on the complexities of assessing pain in horses using automatic methods. We have added this article in the manuscript to present a clearer overall picture of this research topic.

Reviewer #3: I would like to thank the authors for their effort to revise the manuscript according to the reviewers’ suggestions, and generally I am satisfied with the revisions made. I do have, however, some last suggestions and questions. If the authors revise the manuscript accordingly, I will accept the manuscript for publication.

We are very happy to hear that and we would like to thank you for the previous and current revisions, that were extremely helpful to improve the manuscript.

Last suggestions and questions:

L5: Scale with a capital S instead of a small s?

We have corrected it in the Manuscript.

L64: Either ‘a potential threatening stimulus’ or ‘potential threatening stimuli’

We have corrected it in the Manuscript.

L98: Images with a small i instead of a capital I?

We have corrected it in the Manuscript.

L101: space is missing between was and requested. In L103 is a space missing as well.

We have corrected it in the Manuscript.

L129: I would say ‘in 320 videos of 30 minutes each’ instead of ‘in 320 videos each of which was 30 minutes in duration’.

We have corrected it in the Manuscript.

L135: I would like to thank the authors for including the time here. However, the time is equal to the first observation point of the day. I assume that the post-operative procedures were done first and video recordings started thereafter? In addition, the penicillin-based ointment was applied twice a day. When was the second time?

We would like to thank you for this observation. This information was not clear in the manuscript, so we added the observation that the follow ups were carried out before the video recordings in the morning (7am) and in the afternoon (12 pm).

L157/Fig. 2: Although the extra explanation in the figure caption helps, I still find it difficult to see where the mouth and nostrils are in the picture of this parameter in the moderately pain option. I mean for not present and obviously present the nostrils are clearly on the right-side and the mouth on the left, but this is very vague for the in between pain option. Also, in the other two pictures, there is clearly some background beneath the mouth making it clearly standing out from the background/other body parts, but this seems not the case in the picture I have concerns with. If the authors would have a different and clearer picture, that would be great. Otherwise it might work to give even more explanation in the caption regarding e.g. whether the nostrils are on the left or right for instance.

Thank you for this observation. We have changed the image and believe that it is better to understand all the information now.

L170: all instead of both?

We have corrected it in the Manuscript.

L253-254: Here ear and eye in singular, but earlier in plural. Good to make this consistent?

We have corrected it in the Manuscript.

L299: Perhaps add here to the manuscript and not only in reply to the reviewers some words about that this work is “preliminary work that seeks to show the potential of the proposed method. [...] We also aim to spread the word to inspire other groups of scientists who can evolve with the work, and create potential collaborative arrangements.”. In this way the authors show that they are, for instance, aware of the model’s shortcomings at this stage in the process. Thus, that it is a work in process.

Thank you for this observation, we have added this information to the manuscript.

7. PLOS authors have the option to publish the peer review history of their article ([what does this mean?](https://journals.plos.org/plosone/s/editorial-and-peer-review-process#loc-peer-review-history)). If published, this will include your full peer review and any attached files.

If you choose “no”, your identity will remain anonymous but your review may still be made public.

**Do you want your identity to be public for this peer review?** For information about this choice, including consent withdrawal, please see our [Privacy Policy](https://www.plos.org/privacy-policy).

Reviewer #1: No

Reviewer #3: No
