# Supplementary material for: Pain assessment in horses using automatic facial expression recognition through deep learning-based modeling
Source: PLoS One. 2021 Oct 19;16(10):e0258672. doi: 10.1371/journal.pone.0258672 (PMC8525760; doi:10.1371/journal.pone.0258672)
Supplement: Attachment — Submitted filename: Response to reviewers.docx [file pone.0258672.s003.docx]

Dear Dr. Humainara Nisar and reviewers,

Thank you very much for your important considerations, we have addressed all of them and we hope that the manuscript is now acceptable for publication.

In the document below we are describing how we answered the considerations and carried out the suggested changes on the manuscript.

Journal Requirements:

Please review your reference list to ensure that it is complete and correct. If you have cited papers that have been retracted, please include the rationale for doing so in the manuscript text, or remove these references and replace them with relevant current references. Any changes to the reference list should be mentioned in the rebuttal letter that accompanies your revised manuscript. If you need to cite a retracted article, indicate the article’s retracted status in the References list and also include a citation and full reference for the retraction notice.

We reviewed the reference list and we understand that it is complete and correct.

Additional Editor Comments (if provided):

[Note: HTML markup is below. Please do not edit.]

Reviewers' comments:

Reviewer's Responses to Questions

**Comments to the Author**

1. If the authors have adequately addressed your comments raised in a previous round of review and you feel that this manuscript is now acceptable for publication, you may indicate that here to bypass the “Comments to the Author” section, enter your conflict of interest statement in the “Confidential to Editor” section, and submit your "Accept" recommendation.

Reviewer #1: (No Response)

Reviewer #3: All comments have been addressed

2. Is the manuscript technically sound, and do the data support the conclusions?

The manuscript must describe a technically sound piece of scientific research with data that supports the conclusions. Experiments must have been conducted rigorously, with appropriate controls, replication, and sample sizes. The conclusions must be drawn appropriately based on the data presented.

Reviewer #1: Partly

Reviewer #3: (No Response)

3. Has the statistical analysis been performed appropriately and rigorously?

Reviewer #1: N/A

Reviewer #3: (No Response)

4. Have the authors made all data underlying the findings in their manuscript fully available?

The [PLOS Data policy](http://www.plosone.org/static/policies.action#sharing) requires authors to make all data underlying the findings described in their manuscript fully available without restriction, with rare exception (please refer to the Data Availability Statement in the manuscript PDF file). The data should be provided as part of the manuscript or its supporting information, or deposited to a public repository. For example, in addition to summary statistics, the data points behind means, medians and variance measures should be available. If there are restrictions on publicly sharing data—e.g. participant privacy or use of data from a third party—those must be specified.

Reviewer #1: Yes

Reviewer #3: (No Response)

5. Is the manuscript presented in an intelligible fashion and written in standard English?

PLOS ONE does not copyedit accepted manuscripts, so the language in submitted articles must be clear, correct, and unambiguous. Any typographical or grammatical errors should be corrected at revision, so please note any specific errors here.

Reviewer #1: Yes

Reviewer #3: (No Response)

6. Review Comments to the Author

Please use the space provided to explain your answers to the questions above. You may also include additional comments for the author, including concerns about dual publication, research ethics, or publication ethics. (Please upload your review as an attachment if it exceeds 20,000 characters)

Reviewer #1: I think the revised manuscript is acceptable for publication.

Thanks to the authors for their passionate research contribution and professional effort at revising the manuscript in order to address the reviewers' comments.

Nevertheless, I have 3 comments (a minor grammatical comment and two other technical comments) as below:

_______________________________________________________________________________________

Comment 1:

【line 220 in manuscript (or line 225 in manuscript with tracked changes)】

- resulting in (instead of resulting on)

Thank you for your comment. It was corrected in the manuscript.

_______________________________________________________________________________________

Comment 2:

【lines 272-274 in manuscript】

"The final value for the number of neurons in the hidden layer, the learning rate and the momentum hyperparameters were 5, 0.3 and 0.2 respectively."

If it is possible, it could be valuable to report the final weights and biases of the 5 hidden nodes, since the weights and biases of these 5 hidden nodes carry information regarding the relative significance of 3 facial regions and the number of nodes (3 x 5 + 5 = 20 values ?) are not too large too be presentable.

Thank you for your comment.

All generated models and the buffer results were added to the data repository.

Lencioni, Gabriel; Sousa, Rafael; Sardinha, Edson; Romero, Rodrigo; Zanella, Adroaldo (2021), “Automatic Pain Assessment in Horses”, Mendeley Data, V3, doi: 10.17632/t8rtzcgwxm.3

Comment 3 (quite an important query as to the validity of the 10-fold cross validation results):

- Referring to 【lines 217-219】 & 【lines 223-225】 of the manuscript with tracked changes or 【lines 216-218】 & 【lines 219-221】 of the manuscript,

"The Levenberg-Marquardt backpropagation method and mean squared error were employed to measure the performance using k-fold cross validation of 10" and

"Forty complete original images were randomly selected of animals from each class: no pain, moderately present pain and obviously present pain, resulting on 120 complete images."

But the results reported in Table 7 and Table 8 contained all the 120 images and this is not the averaged results of the 10-fold cross validation.

May I know how the 10-fold validation was conducted and why the results reported is not the averaged 10-fold cross-validated result?

Using 120 images for 10-fold cross validation, each fold of validation should have only 12 validation images, not the 120 as reported in Tables 7 & 8. Or has the author presented the summation of the 10-fold cross-validation, instead of the average? This should be clarified in the Table's caption or the content of manuscript to avoid confusion.

Thank you for your comment. We carried out the suggested changes in order to address these issues. We believe that the title of table 7 and 8 could lead to this confusion. We have changed the title of both tables and explained in the methods section that the performance metrics were based on the average values of all the folds. (The performance indicators are calculated by the average values and the table showing the hits and errors is formed by the summation: https://www.cs.waikato.ac.nz/ml/weka/mooc/dataminingwithweka/transcripts/Transcript2-5.txt).

_______________________________________________________________________________________

End of Recommendation

Reviewer #3: (No Response)

7. PLOS authors have the option to publish the peer review history of their article ([what does this mean?](https://journals.plos.org/plosone/s/editorial-and-peer-review-process#loc-peer-review-history)). If published, this will include your full peer review and any attached files.

If you choose “no”, your identity will remain anonymous but your review may still be made public.

**Do you want your identity to be public for this peer review?** For information about this choice, including consent withdrawal, please see our [Privacy Policy](https://www.plos.org/privacy-policy).

Reviewer #1: No

Reviewer #3: No

[NOTE: If reviewer comments were submitted as an attachment file, they will be attached to this email and accessible via the submission site. Please log into your account, locate the manuscript record, and check for the action link "View Attachments". If this link does not appear, there are no attachment files.]

While revising your submission, please upload your figure files to the Preflight Analysis and Conversion Engine (PACE) digital diagnostic tool, <https://pacev2.apexcovantage.com/>. PACE helps ensure that figures meet PLOS requirements. To use PACE, you must first register as a user. Registration is free. Then, login and navigate to the UPLOAD tab, where you will find detailed instructions on how to use the tool. If you encounter any issues or have any questions when using PACE, please email PLOS at figures@plos.org. Please note that Supporting Information files do not need this step.
